# Supplementary material for: Cell Surface Hsp90- and αMβ2 Integrin-Mediated Uptake of Bacterial Flagellins to Activate Inflammasomes by Human Macrophages
Source: Cells. 2022 Sep 15;11(18):2878. doi: 10.3390/cells11182878 (PMC9496951; doi:10.3390/cells11182878)
Supplement: Supplementary file 1 [file cells-11-02878-s001.zip › cells-1869868-supplementary.pdf]

Cell Surface Hsp90- and αMβ2 Integrin-Mediated Uptake of Bacterial Flagellins to Activate Inflammasomes by Human Macrophages

Supplementary data

Table S1. Identification of the interacting partners of the Hsp90 by LC-MS/MS.

	Uniprot Accession number	% coverage	No. of unique peptides	Average molecular mass (Da)	Protein name	Cellular function	Cellular localization
1	P46940 | IQGA1_HUMAN	17	8	189761	Ras GTPase-activating-like protein IQGAP1	Regulation of GTPase activity	Plasma membrane
2	Q0VD83 | APOBR_HUMAN	17	4	115261	Apolipoprotein B receptor	Metabolism, lipid transport	
3	P05107 | ITB2_HUMAN	15	6	87985	Integrin beta-2	Immunity, receptor	
4	P01848 (TRAC_HUMAN)	14	1	7205	T cell receptor alpha chain V-J-region	Receptor	
5	P11142 | P11142_HUMAN	10	3	71082	Heat shock cognate 71 kDa protein isoform 1	Stress response, transcription regulation	
6	P11215 | ITAM_HUMAN	10	3	128410	Integrin alpha-M	Immunity, receptor	
7	Q01518 | CAP1_HUMAN	8	3	51926	Adenylyl cyclase-associated protein 1 isoform a	Actin binding	
8	O75976 | CBPD_HUMAN	7	1	153919	Carboxypeptidase D	Protein processing	
9	P16284 | PECA1_HUMAN	6	3	83382	Platelet endothelial cell adhesion molecule precursor	Cell adhesion	
10	P13796 | PLSL_HUMAN	6	2	70814	Plastin-2	Actin regulation	
11	P05023 | AT1A1_HUMAN	5	1	114135	Sodium/potassium-transporting ATPase subunit alpha-1 isoform a	Ion transport	
12	P08865 | RSSA_HUMAN	6	1	32947	40S ribosomal protein SA isoform 1	Protein processing	
13	Q53H76 | PLA1A_HUMAN	9	1	57065	Phospholipase C	Metabolism	
14	O43175 | SERA_HUMAN	10	5	57365	D-3-phosphoglycerate dehydrogenase	Protein biosynthesis	
15	Q14152 | EIF3A_HUMAN	14	11	166867	Eukaryotic translation initiation factor 3 subunit A	RNA-binding, protein biosynthesis	Cytoplasm
16	P49327 | FAS_HUMAN	9	5	275877	Fatty acid synthase	Metabolism	
17	P13929 | ENOB_HUMAN	9	3	47299	Beta-enolase isoform 1	Metabolism	
18	Q9HD40 | SPCS_HUMAN	9	2	49770	Selenocysteine synthase	Protein biosynthesis	

19	Q01581	HMCS1_HUMAN	8	1	61656	Hydroxymethylglutaryl-CoA synthase, cytoplasmic isoform 1	Metabolism	
20	P28838	AMPL_HUMAN	8	4	54754	LAP3 protein	Proteolysis	
21	P50502	F10A1-_HUMAN	7	1	27561	Hsp70 interacting protein	Protein folding	
22	P23381	SYWC-_HUMAN	7	1	53474	Tryptophan-tRNA ligase, cytoplasmic	Protein biosynthesis	
23	Q99832	TCPH_HUMAN	6	1	59842	T-complex protein 1 subunit eta	Nucleotide binding	
24	Q13085	ACACA_HUMAN	6	2	266469	Acetyl-CoA carboxylase	Metabolism	
25	P35573	GDE_HUMAN	5	2	176819	Glycogen debranching enzyme isoform 1	Glycogen biosynthesis	
26	P35998	PRS7_HUMAN	5	1	49002	26S proteasome regulatory subunit 7 isoform 1	ATP binding	
27	P50991	TCPD_HUMAN	14	6	58401	T-complex protein 1 subunit delta	ATP binding, nucleotide binding	
28	Q7Z406	MYH14_HUMAN	11	5	107357	MYH14 protein	Cytokinesis, cell shape	
29	Q00610	CLH1_HUMAN	9	19	193260	Clathrin heavy chain 1 isoform 1	Cell cycle regulation	
30	P04792	HSPB1_HUMAN	9	1	22826	Heat shock protein beta 1	Stress response	
31	Q14141	SEPT6_HUMAN	8	1	50068	Septin 6	Cell cycle regulation, Differentiation	
32	P0DPH7	TBA3C_HUMAN	8	1	46767	Tubulin alpha 3C	ATP binding, nucleotide binding	
33	P50990	TCPQ-_HUMAN	8	2	60195	T-complex protein 1 subunit theta	ATP binding, nucleotide binding	
34	Q6PEY2	TBA3E_HUMAN	7	1	50568	Tubulin alpha-3E	Nucleotide binding	Cytoskeketon
35	Q13885	TBB2A_HUMAN	7	2	50274	Tubulin beta-2A chain	Nucleotide binding	
36	P35579	MYH9_HUMAN	6	6	227745	Non-muscle myosin heavy chain 9	Cell adhesion	
37	O75369	FLNB_HUMAN	6	1	280157	Filamin B isoform 2	Myogenesis, Differentiation	
38	Q3ZCM7	TBB8_HUMAN	6	2	49841	TUBB8 protein	Nucleotide binding	
39	P04264	K2C1_HUMAN	5	3	58994	Keratin, type II cytoskeletal 1	Protein activity regulation	
40	Q14839	CHD4_HUMAN	5	2	200203	Chromodomain-helicase-DNA-binding protein 4 isoform 1	Chromodomain-helicase-DNA-binding protein 4 isoform 1	
41	P04075	ALDOA_HUMAN	5	1	28789	Fructose-bisphosphate aldolase A	Metabolism	
42	Q00341	VIGLN_HUMAN	15	8	141995	Vigillin	Metabolism	
43	A0A024R4E5	A0A024R4E5_HUMAN	14	3	141995	High density lipoprotein-binding protein	RNA binding	Cytosol
44	P07814	SYEP_HUMAN	8	7	172080	Bifunctional glutamate/proline-tRNA ligase	Translation regulation	
45	P41091	IF2G_HUMAN	7	1	51647	Eukaryotic translation initiation factor 2 subunit 3	Protein biosynthesis	
46	Q96QK1	VPS35_HUMAN	6	1	92507	Vacuolar protein sorting 35 homolog	Protein transport	Endosome
47	Q9Y490	TLN1_HUMAN	15	32	271766	Talin 1	Cell-cell adhesion	

48	P21281 l VATB2_HUMAN	9	1	56807	V type proton ATPase subunit B, brain isoform	Ion transport	Endomembrane
49	O95782 l AP2P1_HUMAN	8	2	109096	AP2A1 protein	Protein transport	
50	P26038 l MOES_HUMAN	7	1	67892	Moesin	Actin cytoskeleton modification	
51	P11021 l BIP_HUMAN	8	23	72402	Endoplasmic reticulum chaperone BiP precursor	Host-virus interaction	Endoplasmic reticulum
52	P50454 l SERPH_HUMAN	8	2	46525	Serpin H1 precursor	Stress response	
53	Q9Y4L1 l HYOU1_HUMAN	8	3	111494	Hypoxia up-regulated protein 1 precursor	Stress response	
54	B3KQT9 l B3KQT9_HUMAN	8	1	59947	Protein disulfide-isomerase	Protein folding	
55	P13667 l PDIA4_HUMAN	7	6	57480	Protein disulfide-isomerase A4 precursor	Molecular chaperone, catalytic enzyme	
56	B4DWK8 l B4DWK8_HUMAN	7	1	28615	Catalase	Metabolism	
57	Q59G92 l Q59G92_HUMAN	5	1	119800	HLA-B associated transcript 1 variant	Immunity	
58	P25705 l ATPA_HUMAN	7	3	23402	ATP synthase subunit alpha, mitochondrial precursor	Ion transport	Extracellular
59	P02768 l ALBU_HUMAN	6	4	71317	Serum albumin	Regulation blood pressure	
60	P13645 l K1C10_HUMAN	5	1	58994	Keratin, type 1 cytoskeletal 10	Structure integrity	
61	A0A024R157 l A0A024R157_HUMAN	42	9	175437	UDP-glucose ceramide glucosyltransferase-like 1	Metabolism	Golgi apparatus
62	A0A024R8M5 l A0A024R8M5_HUMAN	11	5	23402	hCG1818127, isoform CRA_a	Transferase activity regulation	
63	P05091 l ALDH2_HUMAN	7	1	56859	Aldehyde dehydrogenase, mitochondrial isoform 1 precursor	Metabolism	
64	Q08378 l GOGA3_HUMAN	7	1	167765	Golgin subfamily A member 3 isoform 1	Membrane trafficking	
65	O14617 l AP3D1_HUMAN	6	2	126079	AP-3 complex delta subunit	Protein transport	
66	Q8WUI6 l Q8WUI6_HUMAN	6	2	79132	COPG protein	Protein transport	
67	P46459 l NSF_HUMAN	5	1	83113	N-ethylmaleimide-sensitive factor	Protein transport	Lysosome
68	P06280 l AGAL_HUMAN	5	2	49476	Alpha-galactosidase A	Metabolism	
69	Q0QEN7 l Q0QEN7_HUMAN	17	5	56525	ATP synthase subunit beta, mitochondrial precursor	Angiogenesis, ATP biosynthetic process	Mitochondria
70	P10809 l CH60_HUMAN	11	4	61346	Heat shock protein 60	Host-virus interaction	
71	Q99536 l VAT1_HUMAN	8	1	41659	Synaptic vesicle membrane protein VAT-1 homolog	ATPase activity regulation	
72	P55084 l ECHB_HUMAN	7	1	51547	Trifunctional enzyme subunit beta, mitochondrial isoform 1 precursor	Metabolism	
73	P34897 l GLYM_HUMAN	6	1	56414	Serine hydroxymethyltransfe	Metabolism	

					rase, mitochondrial isoform 1 precursor		
74	Q13077|TRAF1_HUMAN	5	1	79649	TRAP1 protein	Nucleotide binding	
75	P07954|FUMH_HUMAN	5	1	54773	Fumarate hydratase	DNA repair	
76	Q9Y230|RUVB2_HUMAN	17	6	51296	RuvB-like 2	Regulation of transcription	
77	P10276|RARA_HUMAN	10	1	63674	Nucleophosmin-retinoic acid receptor alpha fusion protein	DNA binding, receptor	
78	P16150|LEUK_HUMAN	10	1	40297	Leukosialin precursor	T cell function’s regulation	
79	Q14103|HNRPD_HUMAN	10	1	32985	Heterogeneous nuclear ribonucleoprotein D0 isoform c	DNA/RNA binding,	
80	O075643|U520_HUMAN	10	6	56975	SNRNP200 protein	mRNA processing	
81	P14618|KPYM_HUMAN	9	2	56470	Pyruvate kinase	Glycolysis, Translation regulation	
82	P61011|SRP54_HUMAN	8	1	54331	54 kDa protein	Nucleotide binding	
83	Q9BTE3|MCMBP_HUMAN	8	1	83060	Minichromosome maintenance complex component 5	Cell cycle regulation	
84	P27708|PYR1_HUMAN	8	8	245167	CAD protein isoform 1	Pyrimidine biosynthesis	
85	Q7KZ85|SPT6H_HUMAN	8	5	200203	Transcription elongation factor SPT6	Transcription regulation	
86	Q8NC51|PAIRB_HUMAN	8	3	44291	SERPINE1 mRNA binding protein 1	mRNA stability regulation	
87	P49321|NASP_HUMAN	7	3	85471	Nuclear autoantigenic sperm protein isoform 2	Protein transport	Nucleus
88	P78527|PRKDC_HUMAN	7	1	473749	DNA-dependent protein kinase catalytic subunit isoform 1	Immunity, DNA repair	
89	Q6P2Q9|PRP8_HUMAN	7	2	274738	Pre-mRNA-processing-splicing factor 8	mRNA processing	
90	O60610|DIAP1_HUMAN	7	5	141942	Protein diaphanous homolog 1 isoform 1	Actin cytoskeleton regulation	
91	P60709|ACTB_HUMAN	6	1	41321	Actin, beta	Nucleotide binding	
92	P61158|ARP3_HUMAN	6	2	47797	Actin related protein 3, isoform 1	Cilium biogenesis	
93	Q92797|SYMPK_HUMAN	6	1	141915	Symplekin	Cell adhesion, mRNA processing	
94	P40926|MDHM_HUMAN	6	2	35965	Malate dehydrogenase	Metabolism	
95	Q8TEQ6|GEMI5_HUMAN	6	3	104693	GEMIN5 protei	Translation regulation	
96	P13639|EF2_HUMAN	5	3	96246	Elongation factor 2	Protein biosynthesis	
97	P26358|DNMT1_HUMAN	5	1	185388	DNA methyltransferase 1 isoform b	Translation regulation	
98	P55072|TERA_HUMAN	5	1	89950	Transitional endoplasmic reticulum ATPase isoform 1	DNA repair	

99	Q92878|RAD50_HUMAN	5	1	154823	DNA repair protein RAD50	DNA repair
100	Q15021|CND1_HUMAN	5	2	158448	Condensin complex subunit 1	Cell cycle regulation
101	Q9C0C9|UBE2O_HUMAN	5	1	27645	UBE2O protein	Nucleotide binding
102	O15504|NUP42_HUMAN	5	1	205850	Nucleoporin	Protein transport
103	Q96KC8|DNJC1_HUMAN	5	1	256572	DnaJ (Hsp40) homolog, subfamily C	mRNA processing
104	O43491|E42L2_HUMAN	5	1	113032	Band 4.1-like protein 2 isoform a	Cell cycle regulation

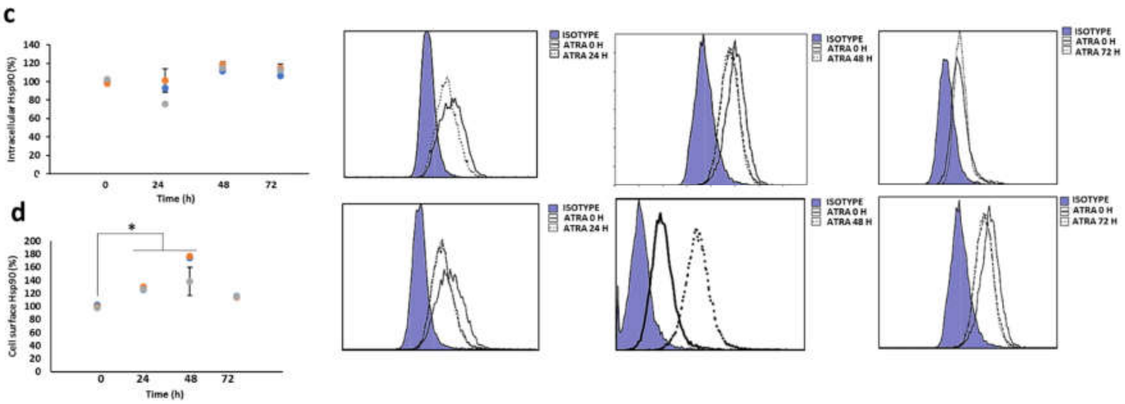

Figure S1. Flow cytometry histogram of figure 1 c, d. THP-1 cells were treated with 1 μM ATRA for various time points. The intracellular (c) or cell surface (d) levels of Hsp90 were measured by flow cytometry. * $p < 0.05$.

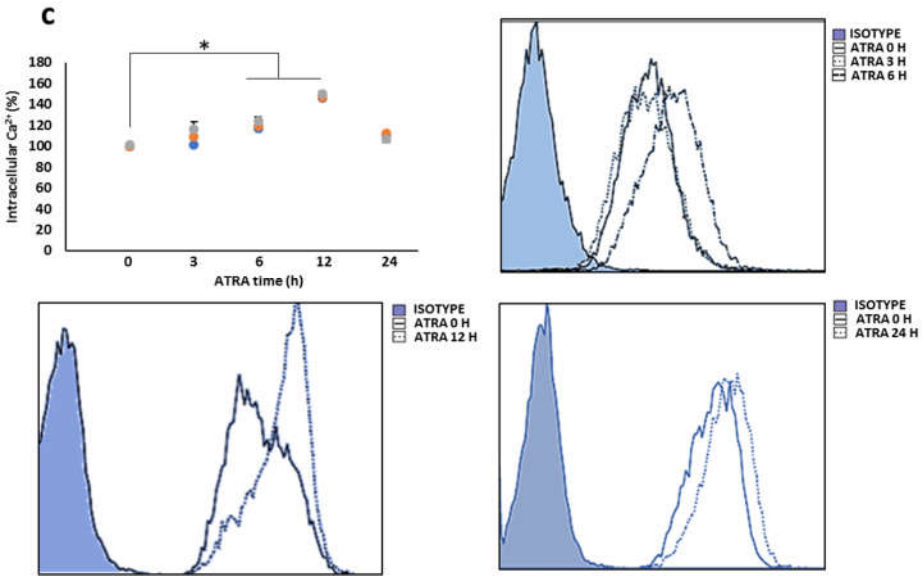

Figure S2. Flow cytometry histogram of figure 3c. THP-1 cells were treated with 1 μM ATRA for various time points as indicated, and the intracellular calcium level was measured by flow cytometry. *$p < 0.05$.

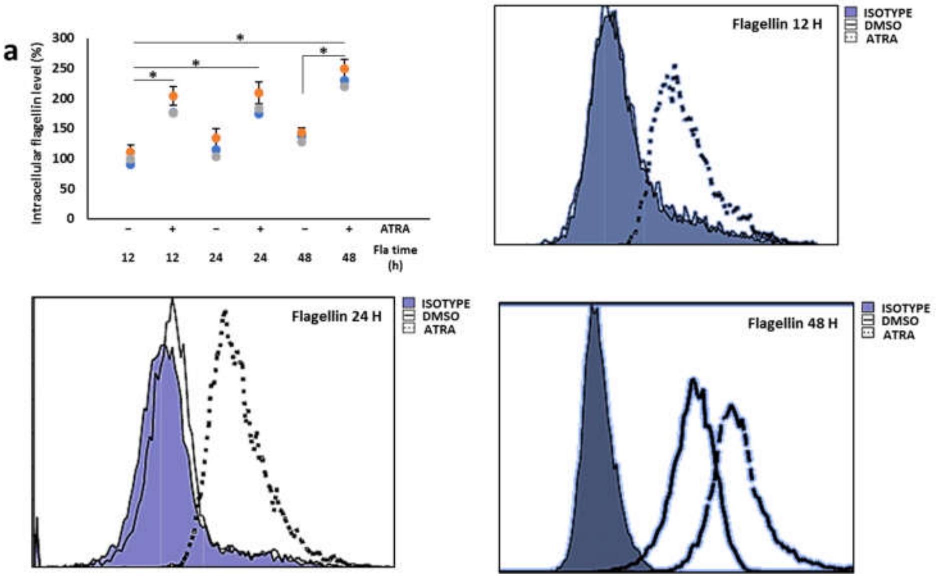

Figure S3. Flow cytometry histogram of figure 6a. THP-1 cells were treated with 1 μM ATRA for 24 h or with DMSO followed by challenge with 100 ng/mL flagellin for different time periods. Intracellular flagellin levels were measured by flow cytometry. *p < 0.05

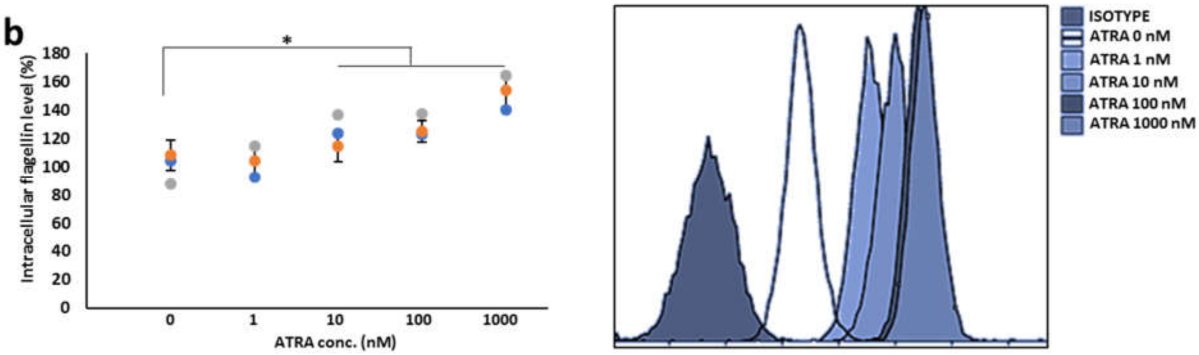

Figure S4. Flow cytometry histogram of figure 6b. THP-1 cells were treated with various concentrations of ATRA for 24 h before 48 h incubation with 100 ng/mL flagellin. Intracellular flagellin levels were measured by flow cytometry. *p < 0.05.

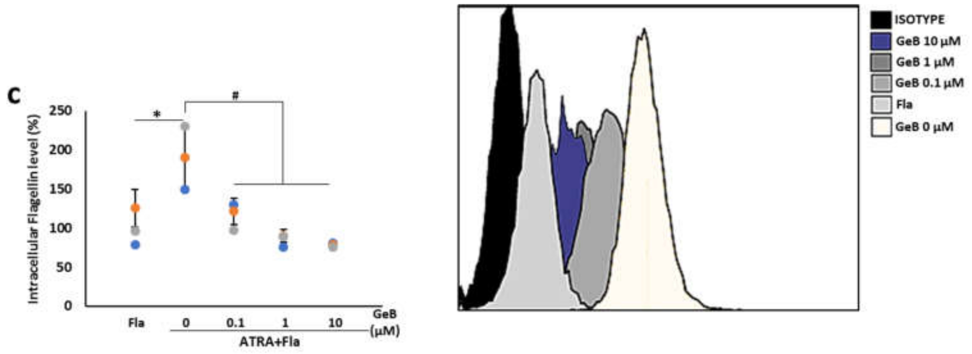

Figure S5. Flow cytometry histogram of figure 6c. THP-1 cells were exposed to different concentrations of GeB for 2 h prior to stimulation with 1 μM ATRA for 24 h and 100 ng/mL flagellin for 48 h. The intracellular level of flagellin was measured by flow cytometry. * $p < 0.05$; # $p < 0.05$.

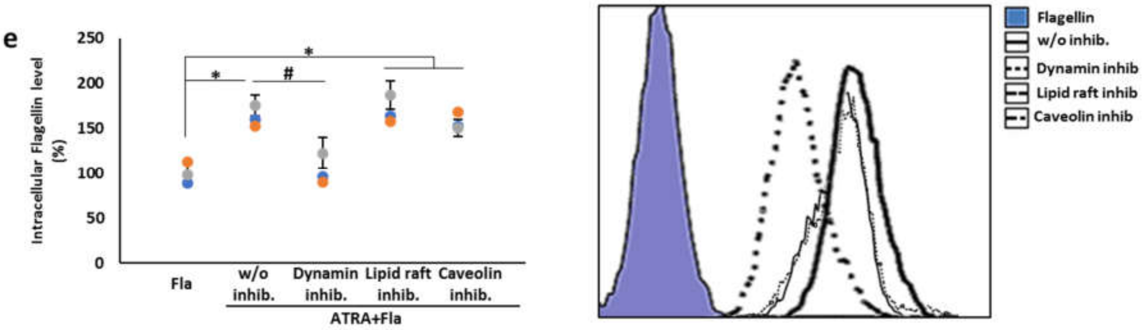

Figure S6. Flow cytometry histogram of figure 6e. THP-1 cells were stimulated with 1 μM ATRA for 24 h prior to treatment with different endocytosis inhibitors (10 μM dynasore (dynamin inhibitor), 5 mM methyl-β cyclodextrin (lipid raft inhibitor) or 1.5 μM filipin III (caveolin inhibitor)) for 2 h. The cells were further treated with 100 ng/mL flagellin for 48 h. The flagellin levels were measured by flow cytometry. * $p < 0.05$; # $p < 0.05$.

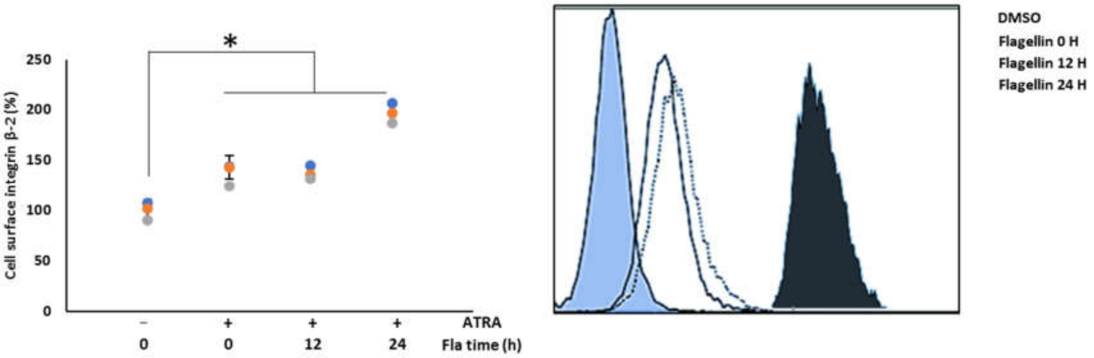

Figure S7. Flow cytometry histogram of figure 7a. THP-1 cells were stimulated with 1 μM ATRA for 24 h followed by treatment with 100 ng/mL flagellin for different time periods, and cell surface integrin β2 was examined by flow cytometry. * $p < 0.05$.

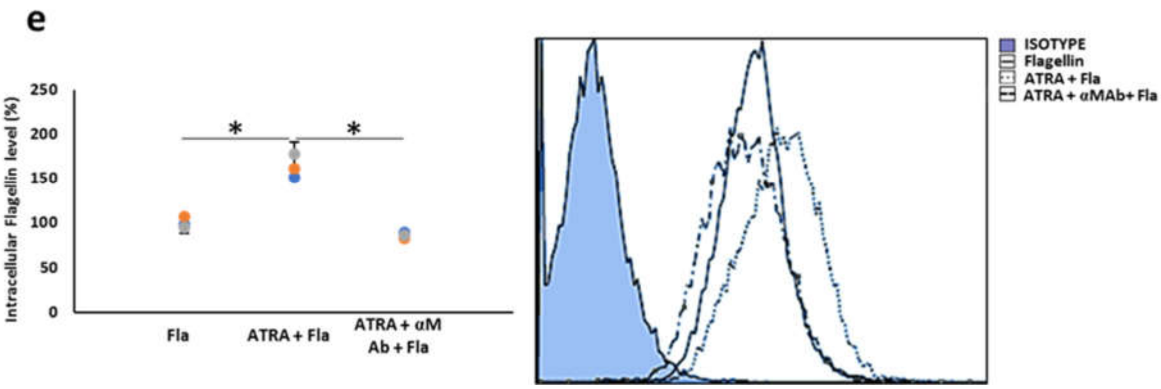

Figure S8. Flow cytometry histogram of figure 7e. THP-1 cells were stimulated with 1 μM ATRA for 24 h, followed by 2 h treatment with 1 μg/mL anti-αM integrin antibody prior to treatment with 100 ng/mL flagellin for 24 h. The intracellular level of flagellin was measured by flow cytometry. * $p < 0.05$.

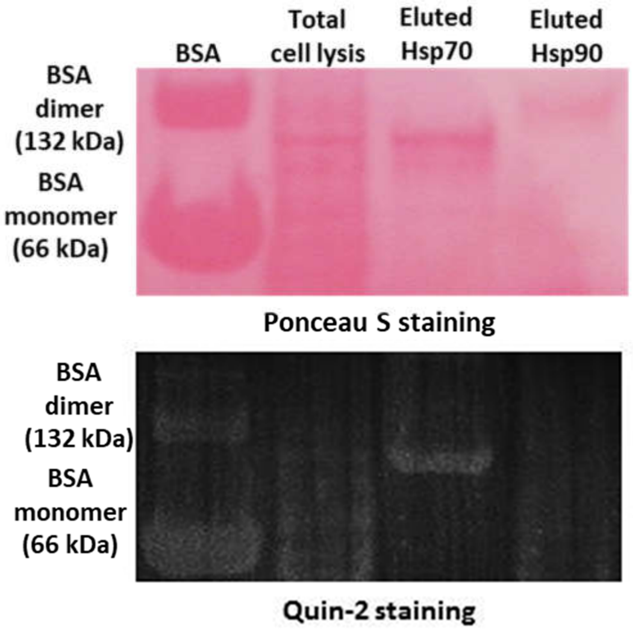

Figure S9. Supplementary data for figure 5f. The proteins were run onto SDS-PAGE followed by membrane transferring. Membrane was then incubated with the developing solution for detecting calcium-binding protein (lower panel) or stained with Ponceau S for protein band visualization (upper panel).
